# The Role of Cardiac Fibroblast Heterogeneity in Myocardial Fibrosis and Its Novel Therapeutic Potential

**DOI:** 10.3390/ijms26125882

**Published:** 2025-06-19

**Authors:** Isotta Chimenti, Francesca Pagano, Claudia Cozzolino, Francesca Icolaro, Erica Floris, Vittorio Picchio

**Affiliations:** 1 Department of Medical and Surgical Sciences and Biotechnologies, Sapienza University of Rome, 00185 Latina, Italy; claudia.cozzolino@uniroma1.it (C.C.); francesca.icolaro@uniroma1.it (F.I.); erica.floris@uniroma1.it (E.F.); 2 Maria Cecilia Hospital, GVM Care & Research, 48033 Cotignola, Italy; 3 Institute of Biochemistry and Cell Biology, National Council of Research (IBBC-CNR), 00015 Monterotondo, Italy; francesca.pagano@cnr.it; 4 Department of Angio Cardio Neurology, IRCCS Neuromed, 86077 Pozzilli, Italy; vittorio.picchio@uniroma1.it

**Keywords:** cardiac remodeling, cardiac diseases, TGF-β signaling, myocardium, single-cell transcriptomics, anti-fibrotic therapies

## Abstract

Cardiac fibrosis is a key physiopathological process underlying the progression of virtually all heart diseases and related conditions, including myocardial infarction, pressure overload, and heart failure. Once regarded as a homogeneous and passive population, cardiac fibroblasts are now recognized as highly heterogeneous and dynamic, comprising distinct subpopulations with specialized molecular and functional identities. These subpopulations include resident fibroblasts, activated myofibroblasts, matrifibrocytes, inflammatory fibroblasts, and senescent fibroblasts, each contributing uniquely to extracellular matrix (ECM) remodeling, cytokine secretion, and intercellular crosstalk. Recent advances in single-cell transcriptomics, lineage tracing, and epigenetic profiling have revealed the plasticity and phenotypic transitions of cardiac fibroblasts in both physiological and pathological contexts. This review synthesizes current knowledge on fibroblast diversity in the adult heart, including their embryological origins and anatomical distribution, and explores how these insights could guide the development of precision anti-fibrotic therapies. We discuss a selection of emerging therapeutic strategies, including subtype-specific targeting (e.g., anti-POSTN, anti-IL1β), modulation of key signaling pathways (e.g., TGF-β, Wnt, Notch), with a brief mention also of novel approaches based on non-coding RNAs and epigenetic regulators. A better understanding of cardiac fibroblast heterogeneity holds significant potential for the design of more specific cell-type and context-tailored interventions, moving toward more effective and personalized treatments for cardiac fibrosis and its sequelae.

## 1. Introduction

### 1.1. Clinical Relevance of Myocardial Fibrosis

Myocardial fibrosis is a pathological process that plays a central role in the pathogenesis and progression of virtually all forms of chronic heart diseases, including hypertensive heart disease, ischemic cardiomyopathy, diabetic cardiomyopathy, and valvular heart disease [1]. At the histological level, fibrosis is characterized by excessive deposition of extracellular matrix (ECM) proteins, particularly collagen type I and III, in the interstitial and/or perivascular spaces of the myocardium [2]. This structural remodeling alters in the long term the biological, mechanical, and electrical properties of the heart, promoting cell stress, increasing myocardial stiffness, impairing contractile (systolic) and elastic (diastolic) functions. These features predispose to altered electrical signals and reduced organ functionality that is leading potentially and progressively toward arrhythmias and heart failure [3].

The extent and pattern of fibrosis are closely associated with clinical outcomes. For instance, diffuse interstitial fibrosis is a strong predictor of all-cause mortality and sudden cardiac death in patients with heart failure [4,5]. As another example, myocardial fibrosis detected by cardiac magnetic resonance imaging was reported as a robust non-invasive biomarker of disease severity and prognosis [6]. Fibrosis burden can be also a key indicator of responsiveness to pharmacologic interventions [7].

From a mechanistic perspective, myocardial fibrosis is not merely a bystander consequence of injury, aiming at filling the gap due to cell death and keeping tissue integrity, but indeed it has to be considered as a dynamic and active process driven by cellular and molecular interactions [8]. Following myocardial injury, either localized such as infarction, or diffuse such as pressure overload or metabolic stress, a reparative response is initiated to maintain tissue integrity. However, when this response becomes persistent or dysregulated, it leads to pathological remodeling characterized by progressive ECM accumulation and loss of functional myocardium [9]. Importantly, fibrosis can develop in the absence of evident cardiomyocyte death, such as in aging or diabetic hearts, indicating that fibrotic signaling pathways may be activated independently of necrosis or apoptosis [10]. Chronic inflammatory signals can obviously play a significant role in activating fibrosis through intercellular crosstalk.

As mentioned above, the clinical implications of fibrosis are multifaceted. First, it contributes to myocardial stiffness, thereby impairing ventricular filling and contributing to diastolic dysfunction. Second, it disrupts the alignment and connectivity of cardiomyocytes, impairing mechanical efficiency and contractility. Third, fibrosis acts as a substrate for electrical conduction abnormalities, promoting potential reentrant arrhythmias such as atrial fibrillation and ventricular tachycardia. These effects contribute and advance in parallel with progressive heart failure, diminished exercise tolerance, and increased hospitalization and mortality rates.

Despite its critical role in cardiac pathophysiology, therapeutic options specifically targeting fibrosis remain limited [11], leaving it as an unmet medical need. Most current treatments, such as renin-angiotensin-aldosterone system (RAAS) inhibitors exert anti-fibrotic effects indirectly. The lack of fibroblast-targeted therapies is partly due to the historical perception of cardiac fibroblasts as a homogeneous and passive population of interstitial cells. However, as recent studies have uncovered, cardiac fibroblasts are remarkably heterogeneous, with distinct subtypes capable of divergent roles in both physiological and pathological settings [12,13,14]. This new awareness underscores the urgent need to dissect more thoroughly the cellular mechanisms driving fibrosis with the potential of identifying novel efficient targets within the fibroblast compartment itself.

Understanding the strong link between the clinical significance of myocardial fibrosis, and its biological and molecular cores not only can shed light on disease progression, but can also highlight a critical gap in translational science and therapeutic strategies. As we move toward a more precise and personalized approach to cardiovascular care, it will be crucial to unravel the complexity of the fibrotic microenvironment and the diverse fibroblast populations that orchestrate it.

### 1.2. Role of Fibroblasts in ECM Deposition

Cardiac fibroblasts are the principal cell type responsible for the synthesis, turnover, and organization of ECM proteins in the myocardium. Under physiological conditions, they maintain tissue homeostasis by producing basal levels of collagens, proteoglycans, and glycoproteins, as well as matrix metalloproteinases (MMPs) and their inhibitors (TIMPs) to regulate ECM remodeling [15]. This balanced activity ensures appropriate structural integrity, biomechanical properties, and electrochemical features to allow coupling among cardiomyocytes.

Following cardiac injury or stress, fibroblasts undergo phenotypic activation and differentiate into myofibroblasts, becoming a proliferative, partly contractile, and highly secretory cell type [16,17]. Myofibroblasts dramatically upregulate the production of interstitial collagens and fibronectin, leading to ECM deposition and fibrotic tissue formation. These activated fibroblasts also contribute in the mid term to scar contraction and wound closure in the damaged area, particularly after a massive, localized injury such as an acute myocardial ischemia. However, their prolonged and/or excessive activation results in adverse remodeling, impaired contractile function, and arrhythmogenesis [18].

Fibroblasts respond dynamically to biochemical cues such as transforming growth factor-beta (TGF-β), interleukin-6 (IL-6), angiotensin II, and endothelin-1, as well as to mechanical stress [19,20]. These stimuli activate intracellular signaling cascades (mainly SMAD, MAPK, and PI3K-AKT pathways), that promote increased profibrotic gene expression. Moreover, the secretory nature of activated fibroblasts includes the increased release of cytokines and mediators enhancing pro-inflammatory signaling, angiogenesis, and cell survival. In fact, fibroblasts secrete paracrine factors that modulate the behavior of neighboring cardiomyocytes, endothelial cells, and immune cells, participating in a complex multicellular response [21].

Despite these known pathways and phenotypic features, recent evidence points to a much more complex functional diversity within the fibroblast population, similarly to the already well-consolidated concept of macrophage polarization. In fact, subpopulations of fibroblasts may exhibit varying capacities for ECM production, responsiveness to cytokines, and interactions with other cell types [21]. For instance, some fibroblast subsets may exhibit anti-fibrotic or reparative properties, producing decorin or fibulin, while others adopt pro-inflammatory phenotypes that may further exacerbate tissue damage by recruiting more leucocytes, promoting more cell stress and death in the tissue [22]. Understanding these functional variations in different cell behaviors and communication is critical for the possible development of novel targeted therapies.

In addition to ECM deposition, fibroblasts are now known to play an integral role also in electrical signaling and conduction within the heart. Through gap junctions and ion channel expression, fibroblasts can influence the electrophysiological behavior of the myocardial syncytium and contribute to arrhythmogenic substrates [23]. This crosstalk underscores the complex roles of fibroblasts beyond matrix production, and highlights their centrality in maintaining and affecting cardiac function overall. Taken together, fibroblasts are not merely passive ECM producers, but dynamic regulators of cardiac homeostasis and pathology. Their role in ECM deposition is certainly complex, although context-dependent, reflecting their capacity for phenotypic plasticity and intercellular communication. Therefore, the challenge appears to be to selectively target maladaptive fibroblast activity while preserving or enhancing their reparative and beneficial trophic functions.

## 2. A Brief Overview of Differential Embryonic Origins and Anatomical Locations

### 2.1. Embryonic Origins

Cardiac fibroblasts are heterogeneous not only in their phenotypic and functional properties, but also in their developmental origins. Lineage tracing studies have revealed that cardiac fibroblasts arise from different embryonic sources, including the epicardium, endocardium, and neural crest [24].

The majority of fibroblasts in the adult heart are indeed derived from the epicardium, the outer mesothelial layer of the embryonic heart. During early development, epicardial cells undergo an epithelial-to-mesenchymal transition (EMT) and migrate deeper into the myocardium, giving rise to fibroblasts and vascular smooth muscle cells [25]. The transcription factor Tcf21 is essential for this lineage commitment and its deletion significantly disrupts fibroblast differentiation. Epicardial-derived fibroblasts predominantly populate the ventricular free wall, thus contributing significantly to the fibroblast pool responsible for ECM homeostasis and fibrotic remodeling in an important part of the organ.

Another distinct subpopulation of cardiac fibroblasts originates from the endocardium, particularly within the atrioventricular cushions and septal regions. These cells more specifically undergo endothelial-to-mesenchymal transition (EndoMT) during embryogenesis and are characterized by expression of markers such as Nfatc1 and VE-cadherin [26,27]. Endocardial-derived fibroblasts are thought to localize preferentially in the interventricular septum and around the valves where they may contribute to regional specific fibrotic responses and valvular diseases [27].

A third, smaller subset of cardiac fibroblasts is derived from neural crest cells, which contribute to the outflow tract and proximal great vessels during early cardiac morphogenesis. Although neural crest-derived fibroblasts represent a minor component in the adult heart, they may retain unique signaling capabilities and epigenetic profiles [28,29]. More studies are needed to understand the correlation and causality of embryonic origin versus acquired diseases during adult life.

The embryonic origin of fibroblasts appears to influence not only their anatomical distribution (discussed in the next paragraph), but also their transcriptomic identity, responsiveness to injury, and profibrotic potential [30]. For example, some studies have shown that epicardial-derived fibroblasts may be more prone to activation and ECM deposition following ischemic injury, while endocardial-derived fibroblasts may exhibit stronger paracrine interactions with endothelial cells and valvular structures [31]. The future integration of fate-mapping studies with specific phenotypic analysis at single-cell level may shed more light on this question. Nonetheless, understanding and integrating the ontogeny of cardiac fibroblasts with other biological and clinical perspectives is crucial to appreciate their diverse roles in cardiac development, repair, and disease. It also provides a context for understanding their heterogeneity observed in single-cell transcriptomic studies, also highlighting the importance of context-dependent fibroblast function.

### 2.2. Anatomical Locations

Cardiac fibroblasts demonstrate notable spatial heterogeneity throughout the heart, with some functional specialization correlating with anatomical distribution. Studies using reporter mouse lines and spatial transcriptomics have revealed that fibroblasts residing in distinct cardiac regions (such as the atria, ventricles, septum, or perivascular zones) display differences in gene expression, proliferative activity, and ECM production [32,33]. For instance, fibroblasts in the atrial myocardium are enriched in genes regulating electrical signaling and may coherently play a role in arrhythmogenesis, while those in the ventricular myocardium exhibit higher expression of structural ECM proteins such as collagen I and III [34], consistent with a stronger biomechanical stimulation in that microenvironment. Perivascular fibroblasts, instead, located around coronary vessels, appear to express distinct profiles of angiogenic factors and regulators of vascular tone, suggesting an adapted role in vessel stability and remodeling consistent with their specific location [35].

Interestingly, regional differences are evident also during disease progression. For example, ventricular fibroblasts exhibit enhanced activation in response to pressure overload, contributing to concentric fibrosis and hypertrophy [36] as a necessary adaptation mechanism. In contrast, fibroblasts in the infarct border zone tend to adopt a pro-inflammatory phenotype, mediating leukocyte recruitment and cytokine secretion [19] consistently with a massive localized necrotic process.

These findings suggest that the anatomical context provides informative signals that shape the microenvironment, thus fibroblast behavior and phenotype. Factors such as local mechanical stress, oxygen tension, and paracrine signals from neighboring cardiomyocytes and endothelial cells likely contribute to regional fibroblast specialization as well [37], as they do for many other cell types.

These fibroblast classification perspectives based on tissue position not only improve our understanding of cardiac remodeling, but have implications also for the design of targeted anti-fibrotic strategies. In fact, interventions that ideally account for regional fibroblast diversity may yield better outcomes than systemic generalized therapies that do not differentiate between functionally distinct fibroblast pools in the same tissue.

## 3. Subpopulations of Cardiac Fibroblasts in Physiological and Pathological Contexts

Advances in single-cell RNA sequencing (scRNA-seq) and integrative transcriptomics have enabled the high-resolution classification of cardiac fibroblasts based on transcriptional and other omics features. Multiple subpopulations with distinct gene expression patterns have been identified in both murine and human hearts, reflecting a continuum of phenotypic states and specialized functions [13,21,38]. From the results of multiple studies, fibroblasts can be transcriptionally grouped into quiescent, matrix-producing, inflammatory, and secretory phenotypes (Table 1). Quiescent fibroblasts express baseline levels of ECM components and remain relatively inactive under homeostatic conditions. Upon stress or injury, they activate profibrotic gene programs, becoming matrix-producing myofibroblasts [17]. Another key axis of classification involves inflammatory fibroblasts, which express high levels of cytokines. Interestingly, emerging studies have also identified fibroblast subsets with even anti-fibrotic potential, which is a completely counter-intuitive function for the traditional view of “filler reparative cells”. These fibroblasts secrete proteoglycans, like decorin, and regulatory molecules that inhibit the master fibroblast-activator, that is TGF-β signaling [32]. Their functional classification is further supported by epigenomic data indicating distinct chromatin accessibility patterns and transcription factor activity across fibroblast subtypes [39].

**Figure 1:**
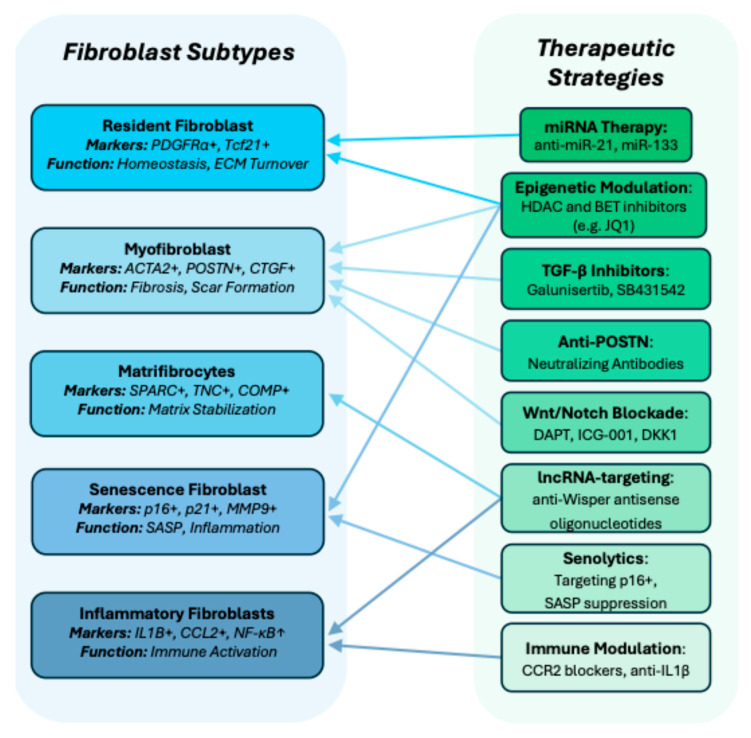
Cardiac fibroblast heterogeneity and therapeutic strategies: The diagram illustrates the heterogeneity of cardiac fibroblast, highlighting distinct fibroblast subtypes, their specific markers, function, and potential therapeutic approaches.

This transcriptomic and functional landscape diversity provides a more precise framework for understanding cardiac fibrosis and potentially opens the door to fibroblast subtype-targeted therapies (Figure 1). Therapeutic strategies aimed at selectively inhibiting profibrotic fibroblast populations, while preserving or enhancing reparative/beneficial subsets, hold promise for future anti-fibrotic approaches. Below we summarize the main features of the different fibroblast subtypes.

### 3.1. Resident Quiescent Fibroblasts

Resident fibroblasts constitute the most abundant non-myocyte cell type in the heart in physiological conditions. These cells are quiescent, spindle-shaped, and express canonical markers such as PDGFRα, Tcf21, and vimentin [20,40]. They synthesize low levels of ECM proteins including collagen type I (gene names of the two main chains: *Col1a1*, *Col1a2*), collagen type III (*Col3a1*), fibronectin (Fn1), and proteoglycans like decorin and biglycan, ensuring matrix homeostasis [9]. Resident fibroblasts also secrete controlled quantities of cytokines (e.g., IL-6) and express balanced ratios of MMPs (MMP2, MMP14) and their inhibitors (TIMP1, TIMP3), supporting normal ECM resorption and turnover [41]. Moreover, they affect other cell types in the microenvironment, including cardiomyocyte and endothelial cells through intercellular communication, both direct and paracrine, but also thanks to matrix-integrin signaling and consequent biomechanical cues [42,43,44].

### 3.2. Myofibroblasts

Activated myofibroblasts occur post injury and are characterized by robust expression of α-smooth muscle actin (α-SMA/*Acta2*) and its polymerization in the cytoplasm, periostin (Postn), and connective tissue growth factor (CTGF) [19,45]. These cells have increased biosynthetic capacity, producing high amounts of ECM proteins and crosslinking enzymes like lysyl oxidase (LOX) [2,43], aiming at consolidating and strengthening the overall ECM structure in the scar tissue. They also secrete abundant pro-fibrotic and pro-inflammatory cytokines (TGF-β1, IL-1β, TNF-α), creating a positive feedback loop that perpetuates fibrosis and inflammation [38]. Through stiffness-induced mechanotransduction and paracrine signals, myofibroblast behavior also alters cardiomyocyte electrophysiology, and impairs angiogenesis and endothelial repair [43,46].

### 3.3. Matrifibrocytes

Matrifibrocytes are a late-stage, matrix-oriented fibroblast subtype that appears after the peak of fibrotic activity and ECM deposition in the scar tissue. They can be identified by the expression of specific proteins, which are Comp, Sparc (osteonectin), and tenascin-C (Tnc). These cells downregulate α-SMA while continuing to stabilize the scar via matrix-interacting proteins and collagen crosslinking activity [47,48]. Although not active cytokine secretors, their role in collagen fibrillogenesis and matrix remodeling is critical for long-term scar integrity [49]. They also interact with all neighboring cell types indirectly through structural and mechanical cues in the remodeled ECM.

### 3.4. Senescent Fibroblasts

Senescent fibroblasts can be detected in response to chronic stress, DNA damage, or oxidative load. They exhibit classical markers of cell cycle withdrawal and replicative senescence, such as p16^INK4a, p21^CIP1, and β-galactosidase activity [50]. Despite having significantly reduced ECM synthesis activity, senescent fibroblasts express elevated levels of MMP9 and acquire a senescence-associated secretory phenotype (SASP), releasing increased amounts of inflammatory mediators like IL-6, IL-8, and MCP-1 (CCL2) [38,51,52]. These secreted factors propagate low-grade inflammation in the myocardium, and may interfere long-term with tissue repair capacity by also altering cardiomyocyte and endothelial survival, stress resistance, and behavior [53].

### 3.5. Inflammatory Fibroblasts

Inflammatory fibroblasts represent a dynamically induced population during acute myocardial damage. These cells express high levels of IL1b, Ccl2, Ccl7, and Cxcl12, and show enrichment for NF-κB-driven transcriptional activity [54]. While not being main producers of ECM, they promote fibrotic signaling by stimulating myofibroblast differentiation [22]. Inflammatory fibroblasts contribute significantly to immune cell recruitment and influence immune phenotypes, especially macrophage polarization and T-cell activation [22]. These cells are critical in modulating immune cell infiltration which can be a significant cause of secondary damage (e.g., for ischemia/reperfusion injury), and have been shown to exacerbate adverse remodeling in models of myocardial infarction and pressure overload. Furthermore, they modulate vascular responses and may impair endothelial barrier function, thereby exacerbating inflammation-driven fibrosis [8,55].

## 4. Therapeutic Implications of Fibroblast Heterogeneity

### 4.1. Targeting Pro-Fibrotic Fibroblast Subpopulations

As already mentioned above, the growing understanding of fibroblast heterogeneity in the heart has prompted a paradigm shift in the potential treatment of cardiac fibrosis: from non-specific suppression of ECM deposition, to the possible targeted modulation of distinct fibroblast subtypes. Among the most well-characterized pro-fibrotic populations are activated myofibroblasts, which express α-SMA, periostin, and CTGF. These cells, derived largely from resident fibroblasts or perivascular cells upon stress or injury, are the major contributors to maladaptive ECM deposition and consequent tissue stiffening.

Several therapeutic strategies have emerged to specifically target myofibroblasts. One approach exploits the unique gene expression profiles of these cells. For instance, *Postn*-driven Cre lines have been used in mouse models to ablate activated fibroblasts selectively, or even deliver toxic genes to fibrogenic subsets [56]. Antibody-mediated neutralization of periostin has also been shown to reduce fibrosis and improve cardiac function post-myocardial infarction in rodents [57].

Cell surface markers like PDGFRα, CD90 (*Thy1*), CD248 (endosialin), and integrin α11β1 are differentially expressed among fibroblast subpopulations, and have been employed for tentative targeted delivery of drugs, nanoparticles, or cytotoxic agents [58,59]. Indeed, anti-CD248 monoclonal antibodies had been explored against fibrotic cancer stroma and are now under evaluation in fibrotic heart tissue.

So called “direct reprogramming” strategies are also under development to convert pro-fibrotic fibroblasts into less harmful or reparative cell types. Introduction of cardiac transcription factors such as GATA4, MEF2C, and TBX5, or microRNAs like miR-1, miR-133a, and miR-208a into cardiac fibroblasts has been shown to induce trans-differentiation into cardiomyocyte-like cells in mouse models, reducing fibrosis and preserving cardiac function [60]. While this approach is still experimental and faces challenges in delivery and efficiency, it holds enormous potential for a combined approach of anti-fibrotic and regenerative medicine.

As another example, selective inhibition of fibroblast migration into the injury site through blockade of chemokines (e.g., CCL2-CCR2 axis) or metalloproteinases has shown anti-fibrotic effects in other organs such as the lung, and could offer a strategy for myocardial fibrosis as well. Ultimately, successful targeting of pro-fibrotic fibroblasts will require a combination of precise biomarkers, selective delivery systems, and real-time monitoring of cell states, especially in human tissues.

### 4.2. Modulation of Key Signaling Pathways

Fibroblast activation is driven by a network of conserved signaling pathways, among which TGF-β, Wnt/β-catenin, and Notch signaling are central to ECM production, myofibroblast differentiation, and intercellular communication. The TGF-β pathway is perhaps the most validated therapeutic target against fibrotic activation. Upon ligand binding, TGF-β1 activates its receptors (TGFBR1/2), leading to phosphorylation of SMAD2/3 and nuclear translocation to regulate genes such as *COL1A1*, *ACTA2*, and *CTGF*. Preclinical studies have shown that inhibition of this pathway using small molecules (e.g., SB431542, LY2157299/galunisertib) or neutralizing antibodies can reduce cardiac fibrosis and preserve ventricular function [16]. However, systemic blockade of TGF-β can lead to severe side effects including impaired immune surveillance and wound healing, therefore this approach will require attentive further evaluation in preclinical studies.

The Wnt/β-catenin pathway is also critical in fibroblast proliferation and survival. Wnt ligands (e.g., Wnt1, Wnt3a) bind Frizzled receptors, inhibit GSK3β, and allow the nuclear translocation of β-catenin, driving transcription of fibrotic targets like *AXIN2*, *POSTN*, and *MMP7*. Small molecule inhibitors such as ICG-001 (β-catenin/CBP inhibitor) and Wnt antagonists like DKK1 or sFRP2, have been shown to reduce ECM accumulation and cardiac dysfunction post MI [61,62], offering promising therapeutic potential.

Notch signaling is also important in fibroblast activation, but it is more context-dependent. Activated by ligands like Jagged1 or DLL4, the Notch intracellular domain (NICD) translocates to the nucleus and induces genes such as *HES1* and *HEY2*. Notch1 activity is increased in cardiac fibroblasts during fibrosis, and promotes proliferation and ECM secretion. Inhibitors such as DAPT (γ-secretase inhibitor) have shown promising results and ameliorated fibrosis in mice in specific models of pressure-overload induction [63], but they will require further assessment for their potential safety and side effects.

The main problem with interfering with the above-mentioned signaling pathways is that, despite being specifically activated in fibroblast subtypes and in a time window for fibrosis, they are nonetheless pleiotropic to other organs and systems. Thus, a key challenge would be the selective targeting of fibroblast-specific signaling axes and timing, while sparing other heterotopic or systemic effects. Approaches under investigation include strategies for fibroblast-restricted gene editing using CRISPR/Cas9 driven by lineage-specific promoters [64], as well as siRNA-based silencing with fibroblast-targeted lipid nanoparticles [65]. Strong efforts will be needed to overcome these many technical obstacles.

### 4.3. Epigenetic and Non-Coding RNA-Based Therapies

Epigenetic regulation governs the plasticity and transcriptional memory of virtually all cell types, including cardiac fibroblasts, making it a compelling target for anti-fibrotic interventions.

Histone deacetylases (HDACs) promote chromatin condensation and silencing of anti-fibrotic genes. HDAC inhibitors such as trichostatin A and vorinostat (SAHA) have been shown to prevent fibroblast-to-myofibroblast transition and enhance transcription of *SMAD7*, an inhibitor of TGF-β signaling [66]. Similarly, inhibitors of bromodomain proteins (e.g., BRD4), like JQ1, can suppress the transcription of pro-fibrotic genes including *MYC* and *NF-κB*-dependent cytokines, leading to reduced inflammation and fibrosis [67]. DNA methylation plays a role in silencing anti-fibrotic genes (e.g., *BMP7*, *SOD2*) as well. Inhibitors like 5-azacytidine and RG108 can demethylate these genes, restoring fibroblast homeostasis toward a quiescent state [68,69]. Again, specificity remains a concern given the pleiotropic nature of these transcriptional modulation pathways.

Non-coding RNAs are emerging as more precise, tissue-specific regulators, and are rapidly advancing toward clinical evaluation as therapeutic molecules [70]. MicroRNAs such as miR-21, miR-155, and miR-199a are known to be upregulated in fibrotic hearts and to promote fibroblast proliferation, ECM deposition, and TGF-β pathway activation. As an example, antagomiRs or miRNA sponges targeting miR-21 have successfully reduced fibrosis in animal models [71], representing another encouraging strategy to target a specific activation mechanism in cardiac fibroblasts.

Other non-coding RNAs are entering the scenario for cardiac fibrosis treatment, though. Long non-coding RNAs (lncRNAs) act as scaffolds or decoys for transcriptional regulators (including microRNAs). Wisper, a lncRNA enriched in cardiac fibroblasts, promotes ECM gene expression by stabilizing transcription complexes at fibrotic loci. Silencing Wisper using gapmer antisense oligonucleotides has been shown to attenuate myocardial fibrosis in vivo [72]. Moreover, other lncRNAs such as Meg3, MIAT, and Neat1 have a role in regulating fibroblast proliferation and cytokine secretion and are being explored as therapeutic targets as well [73,74].

Altogether, these examples of molecular strategies suggest that combining epigenetic and RNA-based interventions with cell-specific delivery platforms could provide high-precision and durable control of cardiac fibroblast activation and differentiation. However, successful clinical translation will require rigorous evaluation of tissue and cell-type specificity, possible immunogenicity or negative effects on the immune system, as well as long-term systemic or off-target effects.

## 5. Conclusions and Future Perspectives

Cardiac fibroblasts are no longer regarded as a uniform and passive structural component of the myocardial stroma. Instead, they are now considered as a highly dynamic and heterogeneous population capable of transitioning between multiple phenotypic states in response to environmental and molecular cues. This concept of fibroblast plasticity (meaning the ability to switch between quiescent, activated, inflammatory, senescent, and even regenerative phenotypes) is fundamental to both cardiac homeostasis and pathological remodeling.

Emerging studies integrating single-cell transcriptomics, lineage tracing, and epigenetic profiling have revealed that fibroblast identity is modulated by the context, the location, and the disease stage. For example, a single fibroblast may transition from a quiescent to an inflammatory state during acute injury, then to a matrix-producing myofibroblast, and eventually to a matrifibrocyte or senescent cell, as scarring and remodeling come to an end at tissue repair. Understanding the signals and transcriptional programs that govern these transitions (e.g., TGF-β, Wnt, Notch, and NF-κB signaling, as well as epigenetic factors like HDACs and non-coding RNAs) will be essential to discovering and designing next-generation therapeutics.

This evolving scenery should lead to the development of precise anti-fibrotic therapies that go beyond global ECM suppression. Ideally, such treatments would target only pathogenic fibroblast subsets (e.g., highly fibrogenic or inflammatory cells) while sparing or enhancing populations that contribute to repair, vascularization, or tissue integrity. The use of cell-type-specific promoters, surface markers, or epigenetic signatures could enable targeted delivery of small molecules, gene editing tools, or RNA-based modulators.

Moreover, temporal specificity will be crucial. Anti-fibrotic interventions may need to be tailored to disease phase: for instance, reducing inflammation early after infarction, while preserving scar stability during long-term healing, or even reversing chronic fibrosis in late-stage heart failure. Such timely precision therapy will probably require the development of specific monitoring or biomarker strategies as well, to follow fibroblast subtype activity in disease progression.

Finally, the integration of multi-omics data from human and animal models, along with AI-driven cell classification or drug prediction, could enhance our capacity to define fibroblast states and their therapeutic susceptibilities. Obviously, translating these insights into the clinic will require the development of adequate delivery systems that are not only cell type-selective, but also preferably cardiac-specific, to avoid off-target effects in other organs where fibroblasts also play essential roles.

In conclusion, a deeper understanding of fibroblast heterogeneity and plasticity is unlocking the potential for precision anti-fibrotic therapies capable of modifying the course of many heart diseases leading to heart failure. This represents a shift from damage limitation to active modulation of the fibrotic microenvironment, with the ultimate goal of improving cardiac function and personalizing treatment for patients with diverse cardiac pathologies.

## Figures and Tables

**Table 1:** Comparative features of cardiac fibroblast subtypes.

Fibroblast Subtype	ECM Production	Cytokine/Growth Factor Secretion	Molecular Markers	Crosstalk with Other Cells
**Resident** **Fibroblasts**	• Moderate • Structural ECM • ECM homeostasis	• Basal IL-6 • TGF-β	• PDGFRα • Tcf21 • Vimentin • Collagen I • Collagen III	• Signal to cardiomyocytes • Modulate endothelial and immune function through paracrine cues
**Myofibroblasts**	• High • Interstitial ECM • Fibrosis and scar	• TGF-β1 • IL-1β • CTGF • TNF-α	• α-SMA • POSTN • CTGF • PDGFRβ	• Influence cardiomyocyte contractility and electrophysiology • Activate endothelial cells and macrophages
**Matrifibrocytes**	• Low-to-moderate • Stabilize ECM • Scar maintenance	Quiescent secretome	• SPARC • COMP • Tenascin-C • Low α-SMA	• Interact with ECM • Indirectly with immune and endothelial cells • Support scar maturation and mechanical stability
**Senescent** **Fibroblasts**	• Low • Elevated MMPs • ECM degradation	SASP phenotype: • IL-6 • IL-8 • MCP-1 • MMPs	• p16^INK4a • p21^CIP1 • SA-β-gal • MMP9	• Recruit immune cells • Impair cardiomyocyte function • Alter endothelial barrier integrity
**Inflammatory** **Fibroblasts**	• Low • Promote remodeling	• High IL-1β • CCL2 • CCL7 • GM-CSF	• IL-1β • CCL2 • CCL7 • CXCL12	• Trigger macrophage activation • Modulate T cell responses • Alter endothelial permeability

**Abbreviations: α-SMA**, Alpha-Smooth Muscle Actin; **CCL2**, Chemokine (C-C motif) Ligand 2; **CCL7**, Chemokine (C-C motif) Ligand 7; **COMP**, Cartilage Oligomeric Matrix Protein; **CTGF**, Connective Tissue Growth Factor; **CXCL12**, Chemokine (C-X-C motif) Ligand 12; **GM-CSF**, Granulocyte-Macrophage Colony-Stimulating Factor; **IL-1β**, Interleukin-1 Beta; **IL-6**, Interleukin-6; **IL-8**, Interleukin-8; **MCP-1**, Monocyte Chemoattractant Protein-1; **MMP9**, Matrix Metallopeptidase 9; **MMPs**, Matrix Metalloproteinases; **p16^INK4a**, Cyclin-Dependent Kinase Inhibitor 2A; **p21^CIP1**, Cyclin-Dependent Kinase Inhibitor 1A; **PDGFRα**, Platelet-Derived Growth Factor Receptor Alpha; **PDGFRβ**, Platelet-Derived Growth Factor Receptor Beta; **POSTN**, Periostin; **SA-β-gal**, Senescence-Associated Beta-Galactosidase; **SPARC**, Secreted Protein Acidic and Rich in Cysteine; **Tcf21**, Transcription Factor 21; **TGF-β**, Transforming Growth Factor Beta; **TGF-β1**, Transforming Growth Factor Beta 1; **TNF-α**, Tumor Necrosis Factor Alpha.

## Data Availability

Not applicable.
